# One hand clapping: lateralization of motor control

**DOI:** 10.3389/fnana.2015.00075

**Published:** 2015-06-02

**Authors:** Quentin Welniarz, Isabelle Dusart, Cécile Gallea, Emmanuel Roze

**Affiliations:** ^1^Neuroscience Paris Seine, CNRS UMR8246, Inserm U1130, Sorbonne Universités, UPMC UM119Paris, France; ^2^Inserm U1127, CNRS UMR 7225, Sorbonne Universités, UPMC UMR S1127, Institut du Cerveau et de la Moelle épinière, ICMParis, France; ^3^Département des Maladies du Système Nerveux, AP-HP, Hôpital Pitié SalpêtrièreParis, France

**Keywords:** mirror movement, hopping gait, corticospinal tract, corpus callosum, spinal cord, axon guidance

## Abstract

Lateralization of motor control refers to the ability to produce pure unilateral or asymmetric movements. It is required for a variety of coordinated activities, including skilled bimanual tasks and locomotion. Here we discuss the neuroanatomical substrates and pathophysiological underpinnings of lateralized motor outputs. Significant breakthroughs have been made in the past few years by studying the two known conditions characterized by the inability to properly produce unilateral or asymmetric movements, namely human patients with congenital “mirror movements” and model rodents with a “hopping gait”. Whereas mirror movements are associated with altered interhemispheric connectivity and abnormal corticospinal projections, abnormal spinal cord interneurons trajectory is responsible for the “hopping gait”. Proper commissural axon guidance is a critical requirement for these mechanisms. Interestingly, the analysis of these two conditions reveals that the production of asymmetric movements involves similar anatomical and functional requirements but in two different structures: (i) lateralized activation of the brain or spinal cord through contralateral silencing by cross-midline inhibition; and (ii) unilateral transmission of this activation, resulting in lateralized motor output.

## Introduction

Lateralization of motor control is required for a variety of coordinated movements, including skilled bimanual tasks and locomotion. To our knowledge, only two conditions are associated with the inability to produce asymmetric movements in mammals: human “mirror movements” and rodent “hopping gait”.

Mirror movements are involuntary symmetrical movements of one side of the body that mirror voluntary movements of the other side. The affected individuals are unable to perform purely unimanual movements and have difficulties to perform tasks requiring independent actions with the two hands such as holding a cup while filling it with water, opening a jar or playing a musical instrument. During these tasks, the effectors produce different motor outputs that are usually bound together by a shared, object-directed goal.

Quadrupedal locomotion is characterized by coordinated, alternating bilateral activation of limb muscles, in which effectors repeatedly produce similar motor outputs in a specific temporal order. A “hopping gait” is a switch from alternate to synchronous activity of the limbs during locomotion that is observed in rodent mutants with impaired axonal guidance.

Here we discuss the neuroanatomical substrates and pathophysiological underpinnings of lateralized motor output through the study “mirror movements” and “hopping gait”. Whereas mirror movements are associated with altered interhemispheric connectivity and abnormal corticospinal projections, abnormal spinal cord interneurons trajectory is responsible for the “hopping gait”. Interestingly, the analysis of these two conditions indicates that the production of asymmetric movements involves similar anatomical and functional requirements but in two different structures, the cerebral cortex and the spinal cord, and it emphasizes the importance of proper commissural axon guidance in this process.

## The “Mirror Movement” Paradigm: Inability to Produce Asymmetric Skilled Hand Movements

**Figure 1:**
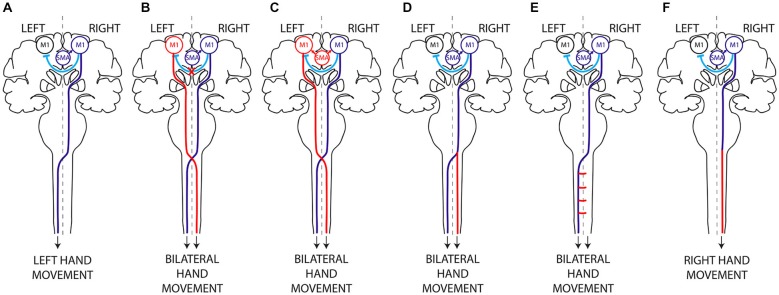
**Hypothetical mechanisms of mirror movements. (A)** In humans, execution of a unilateral left hand movement requires both lateralized activation of the right primary motor cortex (M1) by interhemispheric inhibition (IHI) and proper motor planning and then transmission of the motor command to the contralateral (left) hand alone, through a crossed corticospinal tract. There are two main mechanisms underlying MM: (i) abnormal IHI **(B)** or abnormal delivery of the motor plan from the supplementary motor area (SMA) to M1 **(C)**, resulting in bilateral activation of the primary motor cortices; and (ii) abnormal decussation of the CST **(D)** or abnormal branching of the CST in the spinal cord **(E)**, resulting in bilateral transmission of the motor command to the spinal cord. Mirror movements have not been described in horizontal gaze palsy with progressive scoliosis (HGPPS), despite the absence of CST decussation in these patients **(F)**. This suggests that MM are related to the presence of bilateral spinal cord projections arising from a single primary motor cortex rather than to abnormal decussation of the CST *per se*. Dark Blue, normal mechanism; Red, abnormal mechanism; Light blue, IHI.

Humans have a greater ability than other species to produce purposeful handling movements, most of them being asymmetric. With training, we can master highly complex skills ranging from the fluid movements of the virtuoso pianist to the precise life-saving gestures of the heart surgeon. In humans, execution of unimanual movements requires lateralized activation of the primary motor cortex (M1), which then transmits the motor command to the contralateral hand through the crossed corticospinal tract (CST; Figure 1A; Chouinard and Paus, 2010; Galléa et al., 2011).

Loss of this lateralization results in mirror movements (MM), which consist of involuntary symmetrical movements of one side of the body that mirror voluntary movements of the other side. Congenital mirror movement disorder (CMM) is a rare genetic disorder transmitted in autosomal dominant manner in which mirror movements are the only clinical abnormality. These mirror movements predominate in the distal upper limbs, leaving affected individuals unable to perform independent actions with the two hands or to perform purely unimanual movements. They usually have hand clumsiness and pain in the upper limbs during sustained manual activities. The two main culprit genes are *Dcc* (deleted in colorectal cancer) and *Rad51* (Srour et al., 2010; Depienne et al., 2011, 2012; Méneret et al., 2014a). A third gene, *Dnal4*, might also be involved (Ahmed et al., 2014; Méneret et al., 2014b). *Dcc* plays a key role in CST midline crossing (Finger et al., 2002), while *Rad51* is well known for its role in DNA repair and may also have a major role in motor system development (Depienne et al., 2012; Gallea et al., 2013). In addition to isolated congenital mirror movements caused by *Dcc* or *Rad51* mutations, syndromic forms of MM may be accompanied by numerous other symptoms, in disorders such as Dandy walker syndrome, Joubert’s syndrome, X-linked Kallmann syndrome, Klippel Feil syndrome and congenital hemiparesis (Vulliemoz et al., 2005; Galléa et al., 2011; Peng and Charron, 2013).

CMM provides a unique paradigm for studying the lateralization of motor control (Carson, 2005; Galléa et al., 2011; Peng and Charron, 2013). Two main non exclusive mechanisms may account for MM: (i) abnormal interhemispheric communication resulting in bilateral activation of primary motor areas (Figures 1B,C); and (ii) a corticospinal tract abnormality leading to bilateral downstream transmission of the motor command (Figures 1D,E; Gallea et al., 2013).

### Interhemispheric Connectivity and Motor Lateralization

In humans, the default set-up of motor behavior is probably a mirror program (Chan and Ross, 1988; Meyer et al., 1995; Cincotta and Ziemann, 2008). Unilateral and bilateral voluntary movements are preceded by slow negativity on EEG recordings, known as the *Bereitschaftpotential* (Shibasaki and Hallett, 2006), which starts 2 s before movement onset and is distributed over the two hemispheres. This *Bereitschaftpotential* may reflect bilateral activation of the supplementary motor areas (SMA) and dorsal premotor cortices (dPMC) during motor planning. Just before movement onset, cortical activity is restricted to the primary motor cortex and dPMC contralateral to the intended movement (Shibasaki and Hallett, 2006). An active mechanism is required to restrict motor activation to one hemisphere during execution of a pure unimanual movement.

Our current understanding of this “non mirror transformation” derives mainly from the study of “physiological” mirror movements. Healthy subjects have a default tendency to produce minimal mirror movements when performing highly complex and effortful unimanual tasks (Koerte et al., 2010; Sehm et al., 2010; Beaulé et al., 2012). Activation of the mirror M1 (ipsilateral to the voluntary movement) is the main explanation for this tendency (Mayston et al., 1999; Cincotta et al., 2004; Zijdewind et al., 2006; Hübers et al., 2008). In order to achieve this “non mirror transformation”, the active M1 (contralateral to the intended movement) inhibits the mirror M1 via fibers that pass through the corpus callosum (transcallosal tract, TCT), thereby restricting the motor output to the active M1. This inhibition of one motor cortex by the other is called interhemispheric inhibition (IHI). IHI is thought to rely on transcallosal glutamatergic connections to inhibitory interneurons that in turn innervate pyramidal cells in the receiving hemisphere (Meyer et al., 1995; Reis et al., 2008). Several lines of evidence support the importance of TCT-mediated IHI in the lateralization of motor control. For example, the gradual disappearance of minimal MM frequently observed in young children correlates with the degree of TCT myelination and with the level of IHI (Koerte et al., 2010; Beaulé et al., 2012). Also, experimental modulation of IHI directed from the active M1 to the mirror M1 affects mirror activity: a transient increase in IHI is associated with a decrease in mirror activity, and *vice versa* (Hübers et al., 2008).

IHI between the two primary motor cortices is modulated differently during the different phases of unimanual movements. IHI is balanced between the two motor cortices at the onset of movement preparation, then shifts towards the ipsilateral M1 (ipsilateral to the voluntary movement) at the end of movement preparation and at movement onset (Murase et al., 2004; Duque et al., 2007). In parallel, IHI of the contralateral M1 decreases during movement preparation and shifts towards facilitation at movement onset (Murase et al., 2004; Perez and Cohen, 2008). These subtle time-dependent bilateral variations of IHI are necessary to avoid premature execution (Duque and Ivry, 2009), and to prevent mirror activity in the ipsilateral M1 (Giovannelli et al., 2009). Impairment of IHI may thus result in bilateral M1 activation and transmission of the motor command to both hands through the two crossed CSTs.

In patients with CMM and X-linked Kallmann syndrome, several studies have revealed abnormal, bilateral M1 activation during voluntary unimanual movements and have confirmed that activation of the mirror M1 is not a sensory consequence of the mirror movement but rather participates actively in the mirroring motor activity (Shibasaki and Nagae, 1984; Cohen et al., 1991; Mayer et al., 1995; Cincotta et al., 1996; Krams et al., 1997; Verstynen et al., 2007). However, studies based on indirect methods have failed to demonstrate consistent impairment of IHI mechanisms in CMM patients (Cincotta et al., 1996, 2002; Papadopoulou et al., 2010). Using dual-site transcranial magnetic stimulation (TMS), a more direct method (Perez and Cohen, 2008), we found that CMM patients with *Rad51* mutations had abnormal IHI between the primary motor cortices at rest, together with morphological abnormalities of the TCT (Figure 1B; Gallea et al., 2013). It has been proposed that this impaired IHI is due to an abnormal input of the transcallosal glutamatergic connections onto the inhibitory interneurons in the receiving hemisphere. It is noteworthy that most individuals lacking a corpus callosum do not exhibit mirror movements, suggesting that the absence of the corpus callosum and interhemispheric connections alone might not be sufficient to generate MM. Finally, a study of a CMM patient with complete agenesis of the corpus callosum concluded that the absence of TCT played little part in the pathophysiology of MM (Lepage et al., 2012).

Interhemispheric pathways are not limited to direct M1-M1 interactions and IHI but also include circuits linking secondary motor areas (SMA and PMd) to contralateral motor areas. These circuits might be involved in restricting the generation of motor output to the active hemisphere during movement preparation. For these reasons it has been proposed that abnormal motor planning and/or abnormal transmission of the motor plan from the secondary motor areas to the primary motor areas might also be involved in MM generation (Chan and Ross, 1988; Cincotta et al., 2004; Duque et al., 2010; Galléa et al., 2011; Gallea et al., 2013). Evidence of abnormal motor planning associated with MM was first obtained through studies of two CMM patients and a patient with Kallmann’s syndrome, who showed an abnormal, bilateral (instead of unilateral) distribution of the *Bereitschaftpotential* during movement preparation (Shibasaki and Nagae, 1984; Cohen et al., 1991). However, two other studies argued against a role of abnormal movement planning in MM: the first showed that movement-related cortical EEG potentials were identical (that is to say, lateralized and not bilateral) in healthy volunteers and in six CMM patients (Mayer et al., 1995), while the second study, a case report, showed normal, unilateral cortical activation during fMRI imaging of imagined movements closely related to motor planning (Verstynen et al., 2007). More recently, we found that the SMA activation pattern and connectivity are abnormal during both unimanual and bimanual movements in *Rad51*-mutated CMM patients (Figure 1C; Gallea et al., 2013) This suggested that cortical activation and connectivity might be modified in CMM patients during movement preparation, resulting in inappropriate delivery of the motor program from the SMA to both primary motor cortices.

Together, these results suggest that interhemispheric connectivity is critical for lateralized activation of the motor cortex when a unilateral movement is intended.

### The Corticospinal Tract and Motor Lateralization

The CST is a crossed tract that transmits the motor command from one motor cortex to the contralateral spinal cord. The CST first appeared in mammals and was likely critical for the development of voluntary skilled movements through evolution (Vulliemoz et al., 2005). Selective lesions of the CST in humans, non human primates and rodents impair skilled digit movements such as reaching (Schieber, 2007). The CST is massively crossed in humans. About 70–95% of all CST axons cross the midline at the junction between the medulla and the spinal cord, forming the so-called “pyramidal decussation”, and establish direct contacts with the motor neurons located in the anterior horn of the spinal cord (Vulliemoz et al., 2005). The approximately 10% of CST axons that do not decussate at the medulla remain ipsilateral, and this ipsilateral tract is mainly located in the ventral part of the spinal cord in both humans and rodents (Brösamle and Schwab, 1997; Vulliemoz et al., 2005). The ipsilateral CST component does not target motor neurons innervating distal limb muscles but rather motor neurons innervating the proximal or axial musculatures (Bawa et al., 2004; Vulliemoz et al., 2005). In humans, cats and rodents, the CST initially establishes strong bilateral projections to the spinal cord. The ipsilateral projections consist of uncrossed CST axons (Joosten et al., 1992; Brösamle and Schwab, 1997; Eyre et al., 2001), and/or of normally crossed CST axons that recross the midline within the spinal cord (Li and Martin, 2000; Rosenzweig et al., 2009). This CST projection pattern is refined during early post-natal development, resulting in the elimination of the majority of the ipsilateral projections (Joosten et al., 1992; Eyre et al., 2000, 2001; Li and Martin, 2000). This refinement of the ipsilateral projections is an activity-dependent process of competition with the crossed CST fibers originating from the contralateral motor cortex (Martin and Lee, 1999; Eyre et al., 2001; Eyre, 2003; Martin et al., 2004; Friel and Martin, 2007; Friel et al., 2014).

Human MM could result from the presence of CST projections to both the ipsilateral and contralateral spinal cord. In patients with CMM, Kallmann syndrome, Klippel-Feil syndrome or congenital hemiparesis, unilateral stimulation of the primary motor cortex hand area at rest by TMS elicits bilateral hand muscle responses with identical latencies, whereas in healthy volunteers the muscle response is strictly contralateral to the stimulated hemisphere (Nass, 1985; Farmer et al., 1990; Benecke et al., 1991; Mayston et al., 1997; Alagona et al., 2001; Staudt et al., 2002; Cincotta et al., 2003; Bawa et al., 2004; Srour et al., 2010; Depienne et al., 2011; Gallea et al., 2013). This reveals the presence of fast-conducting corticospinal projections from the hand area of one primary motor cortex to both sides of the spinal cord in CMM patients and suggests an anatomic-functional link between anomalies in the CST trajectory and the inability to produce lateralized movements.

Bilateral corticospinal projections to the spinal cord could be due to: (i) abnormal pyramidal decussation resulting in an aberrant uncrossed ipsilateral CST (Figure 1D); or (ii) aberrant branching of crossed CST axons in the spinal cord (Figure 1E). In both cases, the aberrant CST projection pattern could result from abnormal guidance of the CST axons or from an abnormal persistence of the ipsilateral CST projections that are normally eliminated during development. An elegant TMS study of two CMM patients supports the existence of a separate uncrossed ipsilateral CST (Cincotta et al., 2003). Diffusion tensor imaging (DTI) was used to study the precise anatomy of the pyramidal decussation in *Rad51*-mutated patients, confirming abnormal CST decussation (Gallea et al., 2013), although *Dcc*-mutated CMM patients have yet to be studied. *Rad51* expression pattern in the mouse central nervous system (Depienne et al., 2012), and the known role of *DCC* in commissural axons guidance (Kennedy et al., 1994; Serafini et al., 1994; Keino-Masu et al., 1996; Finger et al., 2002), suggest that abnormal axonal guidance rather than impaired CST maturation is responsible for the bilateral CST projections observed in *Rad51-* and *Dcc*-mutated patients. Electrophysiological studies also support the existence of an aberrant uncrossed CST in X-linked Kallmann patients (Mayston et al., 1997; Farmer et al., 2004). In patients with congenital hemiparesis, MM may be explained by an abnormal maturation of the CST due to the unequal activity between the affected and unaffected motor cortices (Eyre et al., 2001; Eyre, 2003; Friel et al., 2014). This would lead to the maintenance and reinforcement of the ipsilateral CST from the unaffected motor cortex, combined with aberrant branching of corticospinal fibers in the spinal cord (Benecke et al., 1991; Alagona et al., 2001; Staudt et al., 2002; Galléa et al., 2011; Friel et al., 2014). Mirror movements have not been described in patients with horizontal gaze palsy with progressive scoliosis (HGPPS), despite their lack of CST decussation. HGPPS is linked to mutations in the axon guidance receptor ROBO3 (Jen et al., 2004). The CST is completely uncrossed in HGPPS patients, and each hemisphere thus projects in a strictly ipsilateral manner to the spinal cord (Figure 1F). Together, these findings suggest that MM are related to the presence of bilateral spinal cord projections arising from a single primary motor cortex rather than to abnormal decussation of the CST *per se*.

Study of MM patients enlightened the critical importance of two mechanisms for the generation of asymmetric movements: (i) lateralized activation of the brain through contralateral silencing by IHI and proper motor planning; and (ii) unilateral transmission of the motor command to the contralateral spinal cord via the CST. Both abnormal interhemispheric connectivity and an altered CST trajectory could be responsible for MM, but the respective importance of each factor is unclear.

## Control of Left-Right Alternation During Locomotion: New Insights from Genetically Modified Mice with Developmental Motor System Anomalies

Quadrupedal locomotion requires repeated coordinated activity of each limb in a specific temporal sequence. Alternating left-right activity of the forelimbs and hindlimbs is observed at low locomotor frequencies (walking and trotting), while synchronized activity of the homologous limbs is observed at high locomotor frequencies (galloping) in mice, cats, horses and dogs (Forssberg et al., 1980; Dickinson et al., 2000; Serradj and Jamon, 2009). Lateralized motor output is thus a crucial aspect of locomotion, especially at low motor frequencies. In the past decade, careful analysis of genetically modified mice with a “hopping gait” has shed light on the respective contributions of the corticospinal tract and spinal central pattern generators (CPG) to left-right alternation during mouse locomotion.

### The Corticospinal Tract and Left-Right Alternation During Locomotion

**Figure 2:**
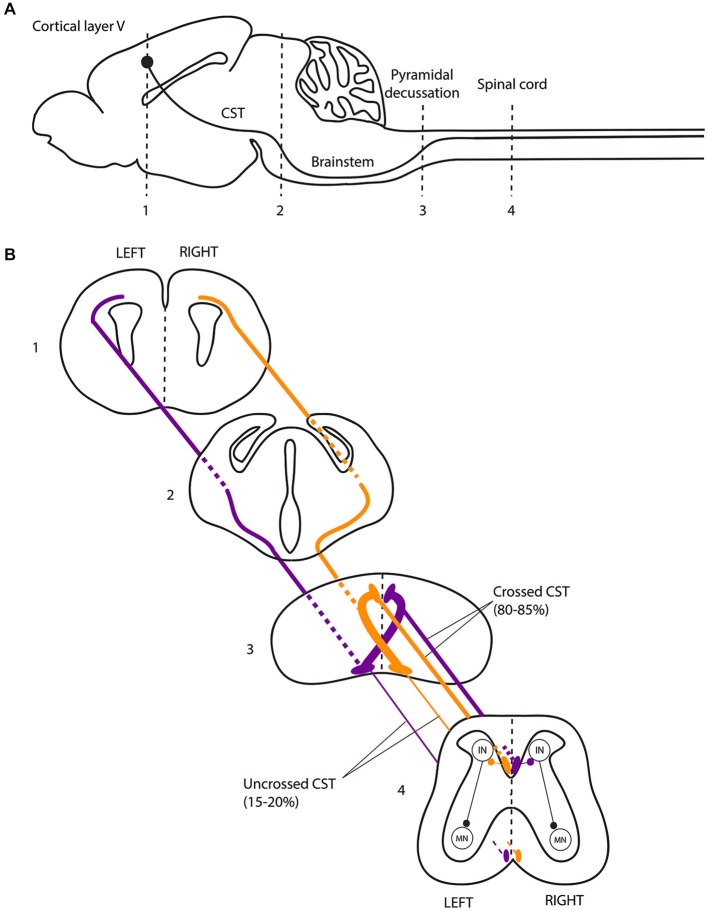
**The corticospinal tract forms a crossed motor system in mice. (A)** Sagittal view of the mouse central nervous system and corticospinal tract (CST). **(B)** Coronal views of the CST trajectory. The level of each coronal schematic section is indicated in figure A. At the junction between the hindbrain and the spinal cord (pyramidal decussation, level 3), the vast majority (80–85%) of corticospinal tract (CST) axons cross the midline and continue their trajectory through the most ventral part of the dorsal funiculus within the half of the spinal cord contralateral to their hemisphere of origin. In the spinal cord, the CST undergoes collateral branching principally at the level of the cervical and lumbar enlargement, eventually transmitting motor commands to the forelimb and hindlimb muscles, respectively, via a multisynaptic pathway involving interneurons mainly located in the dorsal horn of the spinal cord. CST, corticospinal tract; IN, interneurons; MN, motor neurons.

The CST forms a crossed (lateralized) motor circuit controlling voluntary movements of the four limbs. In rodents, the CST is composed of neurons originating from cortical layer V, projecting mainly to the contralateral side of the spinal cord and eventually connecting to motor neurons via a multisynaptic pathway (Figure 2; Canty and Murphy, 2008). A role of the CST in the control of alternating left-right activity during mouse locomotion was initially suggested by the “hopping gait” described in mice with genetically induced alterations of CST projections (mice with mutations of the EphA4 signaling pathway and kanga mice). EphA4 (a member of the Eph family of tyrosine-kinase receptors) and its ligand ephrinB3 are involved in axonal guidance of the CST during development. Deletion of EphA4, Ephrin-B3 or proteins involved in the EphA4 downstream signaling pathway (α2-chimaerin, Nck, RhoA) results in a hopping-gait phenotype (Dottori et al., 1998; Kullander et al., 2001a,b; Yokoyama et al., 2001; Beg et al., 2007; Fawcett et al., 2007; Iwasato et al., 2007; Mulherkar et al., 2013). In EphA4 and EphrinB3 knockout mice, the CST trajectory is normal from the cortex to the pyramidal decussation. In the spinal cord, CST axons re-cross the midline, resulting in bilateral innervation of the spinal cord by each of the two hemispheres. In wild-type animals, EphA4-expressing CST axons are repelled by ephrin-B3 secreted at the midline, deterring them from re-crossing the midline at the spinal level (Dottori et al., 1998; Kullander et al., 2001a,b; Yokoyama et al., 2001). These findings suggested that the hopping gait might be explained by transmission of motor commands to both sides of the spinal cord through abnormally re-crossed CST axons. Similarly to mice with genetic alterations of the EphA4 signaling pathway, a mutant mouse line carrying a viable mutation of the DCC receptor have a “kangaroo-like” hopping gait phenotype and are thus named “kanga” (Finger et al., 2002). The DCC ligand Netrin-1 belongs to the netrin family of extracellular guidance molecules. Netrin-1 has an attractive effect on growth cones when it interacts with the DCC receptor (Keino-Masu et al., 1996). This attraction allows commissural axons to approach and cross the midline (Kennedy et al., 1994; Serafini et al., 1994). DCC is expressed within the main forebrain descending tracts during their development (Shu et al., 2000). In kanga mice, the CST fails to cross the midline at the pyramidal decussation and projects exclusively to the ipsilateral side of the spinal cord (Finger et al., 2002).

However, other experimental findings do not support a major contribution of the CST to alternating left-right activity during locomotion. Indeed, abnormal CST midline crossing is not systematically associated with synchronized activity of the limbs during locomotion: mutants for L1 (Cohen et al., 1998; Jakeman et al., 2006), NCAM (Rolf et al., 2002), Sema6A and PlexinA3/PlexinA4 (Faulkner et al., 2008; Runker et al., 2008), exhibit normal locomotion despite having an abnormal CST. In rodents, a lateralized lesion of the cortex or CST, occurring during the first week of life, leads to sprouting of the remaining CST across the midline and thus to bilateral spinal cord projections (Leong and Lund, 1973; Kartje-Tillotson et al., 1987). This results in altered skilled forelimb movements without affecting left-right alternation during locomotion (Kunkel-Bagden et al., 1992; Whishaw et al., 1993; Whishaw, 2000; Metz and Whishaw, 2002; Tennant and Jones, 2009). Thus, abnormal CST projections do not necessarily induce a hopping gait.

It is important to recall that the genetic alterations induced in EphA4, ephrin-B3 and DCC kanga mutant mice not only impact CST development but also affect commissural cell populations expressing these proteins, such as pre-cerebellar commissural neurons (Hashimoto et al., 2012), and commissural spinal cord interneurons (Kullander et al., 2003; Beg et al., 2007; Iwasato et al., 2007; Rabe Bernhardt et al., 2012). This implies that the hopping gait observed in these mice is not necessarily due to their CST abnormalities. Two recent studies took advantage of the conditional knockout mouse Emx1::cre;EphA4^flox/flox^ in which genetic deletion of EphA4 is restricted to the forebrain. These mice exhibit normal stereotypical locomotion despite bilateral CST projections to the spinal cord (Borgius et al., 2014; Serradj et al., 2014). Together, these results show that proper CST wiring is not necessary for stereotypic left-right alternation.

Supra-spinal control plays a critical role in voluntary movements and adaptive locomotion when sensory-motor integration is required (for example when stepping over an obstacle). Emx1::cre;EphA4^flox/flox^ mice with bilateral CST projections to the spinal cord exhibit symmetric voluntary movements under conditions when asymmetric limbs movements are normally produced (Borgius et al., 2014; Friel et al., 2014; Serradj et al., 2014). These results emphasize the role of the CST in voluntary asymmetric movements.

In addition to the CST, supra-spinal structures playing an important role in the control of gait are located in the cerebral cortex, the cerebellum and in the brainstem, and constitute an interconnected network. There is no clear evidence implicating a supra-spinal control for left-right alternation and lateralization of motor control during gait. Among the locomotor centers with direct spinal projections, the mesencephalic locomotor region (MLR) is of particular interest for our purpose. Electrical stimulation of the brainstem in decerebrate cats placed on a treadmill recapitulates normal alternate locomotion without the need of descending commands from the cortex (Shik et al., 1966, 1967). The MLR, which comprises the pedunculopontine (PPN) and cuneiform (CN) nuclei, sends outputs to the basal ganglia, the cerebellar and the cerebral locomotor areas. The MLR plays a major role in gait initiation and in internal generation of adaptive lower limb movement during the automated gait cycle (Alam et al., 2011; Grabli et al., 2012). The MLR could be involved in the control of gait cadence (Piallat et al., 2009; Karachi et al., 2010), but this involvement is more likely related to higher-order functions during faster gait rather than basic motor control as suggested by rodent models (Winn, 2008). Dysfunction of the MLR and cerebral locomotor centers is observed in patients with Parkinson disease and freezing of gait (Fling et al., 2013, 2014), which is the inability to move the feet despite the effort to overcome the motor block and move forward. These patients exhibit alteration of gait rhythm, gait symmetry and bilateral coordination in stepping (Plotnik and Hausdorff, 2008; Plotnik et al., 2008). However, freezing of gait and bilateral coordination problems are triggered by particular circumstances, when adaptive locomotion is needed (Grabli et al., 2012). In addition, freezing also occurs during writing and speech, although the MLR is not involved in such tasks. MPTP monkeys with selective loss of cholinergic neurons in the PPN have gait impairments but no specific problem in the alternation of lower limb movements (Karachi et al., 2010). In cats, electrical stimulation of the PPN suppresses postural muscle tone, whereas CN stimulation elicits locomotor movements (Takakusaki et al., 2003). In humans, activity of the PPN seems to be modulated during rhythmical stepping, but the increased demands of postural control and attention during stepping could not be cancelled out (Fraix et al., 2013). Therefore, the structures constituting the MLR might play different roles in gait control, but none of them is known to be specifically involved in left-right alternation of the lower limbs.

In non mammalian vertebrates the descending motor pathways are mainly composed of reticulospinal tracts originating from the hindbrain (Vulliemoz et al., 2005). In zebrafish, descending motor pathways include Mauthner cells and other reticulospinal neurons (MiD2cm, MiD3cm and MiD3cl). This crossed network plays a critical role in adaptive locomotor activity such as escape behavior: a stimulus delivered to one side of the head results first in tail bending towards the opposite side, followed by a counter-bend that enables efficient propulsion (Kohashi and Oda, 2008; Jain et al., 2014). *DCC* mutations, leading to midline guidance defects of MiD2cm and MiD3cm neurons that project bilaterally instead of contralaterally, cause an abnormal counter-bend in the same direction as the first. Escape behavior of these mutant zebrafish is thereby compromised. This phenotype is rescued by ablation of the aberrantly projecting MiD2cm and MiD3cm neurons, demonstrating that supra-spinal pathways predominate over spinal circuitry during adaptive locomotion (Jain et al., 2014).

Altogether, these results suggest that supra-spinal control plays a critical role in motor lateralization during voluntary movements and adaptive locomotion but is not involved in left-right alternation during stereotypic locomotion.

### Spinal Control of Left-Right Alternation During Locomotion

The importance of local spinal circuitry in locomotion is supported by “fictive locomotion” experiments performed *in vitro*. Exposure of isolated rodent spinal cords to neurotransmitter agonists such as serotonin and dopamine produces rhythmic activity at the lumbar level lasting several hours. This activity is characterized by alternating ipsilateral flexor-extensor activity and alternating left-right activity (Smith and Feldman, 1987; Kiehn and Kjaerulff, 1996). Successful replication of left-right alternation in spinal cords isolated from the forebrain strongly suggests that the spinal neuronal network plays a critical role in locomotion. This network is called the central pattern generator (CPG), and its role in swimming and walking has been extensively studied (Grillner, 2003; Goulding, 2009; Kiehn et al., 2010; Kiehn, 2011). The CPG generates rhythm, ipsilateral flexor-extensor alternation, and left-right alternation. Spinal commissural interneurons (CIN), mostly located in the ventromedial spinal cord (lamina VIII), play a key role in left-right alternation (Stokke et al., 2002). Fictive locomotion experiments *in vitro* have shown that removal of the dorsal part of the spinal cord does not affect left-right alternation, whereas sectioning of the ventral spinal cord commissure completely abolishes it (Kjaerulff and Kiehn, 1996). When inhibitory GABAa or glycinergic CIN are neutralized by the use of antagonists, spinal left-right alternating activity switches to synchronous activity, demonstrating that this cross-midline inhibition is critical for lateralized motor activity (Cowley and Schmidt, 1995). Conversely, suppression of glutamatergic excitatory transmission in the spinal cord of Vglut2 mutants does not affect the generation of left-right alternation or locomotor rhythms (Gezelius et al., 2006; Wallén-Mackenzie et al., 2006).

The specific characteristics and fate of spinal cord interneurons are determined by the progenitor subtype from which they originate. During the early phases of CNS development, transcription-factor gradients result in dorsoventral patterning of spinal neurons. There are 11 progenitor domains in the spinal cord, six dorsal (dI1-dI6) and five ventral (V0, V1, V2, motor neurons and V3 interneurons, in dorsal-to-ventral order; Jessell, 2000; Arber, 2012). Delineation of the CPG circuitry through the use of mutant mice improved our understanding of spinal alternating left-right activity.

**Figure 3:**
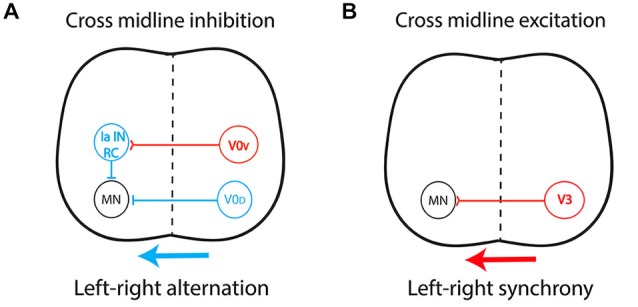
**Central pattern generator circuitry underlying left-right coordination during locomotion. (A)** The dual inhibitory pathway (glycinergic/GABAergic) is composed of V0-derived interneurons including: (i) a group of dorsal inhibitory commissural interneurons (CIN) that project monosynaptically to contralateral motor neurons (V0_D_); and (ii) a group of ventral glutamatergic CIN (V0_V_) which provide indirect inhibition via a multisynaptic pathway with ipsilateral inhibitory interneurons such as Renshaw cells (RC) and Ia inhibitory interneurons (Ia IN). This system produces cross-midline inhibition that allows contralateral silencing during left-right alternation, in a frequency-dependent manner. **(B)** The excitatory pathway is composed of glutamatergic CIN (V3) projecting directly to contralateral motor neurons, providing the support for left-right synchronicity. Red, excitatory interneurons; Blue, inhibitory interneurons.

By connecting the two sides of the spinal cord, CIN determine the excitatory/inhibitory balance over the midline. Most CIN involved in left-right coordination originate from the ventral spinal cord, from V0 and V3 progenitors (Kiehn, 2011; Chédotal, 2014), but the role of dorsally derived interneurons was recently highlighted (Andersson et al., 2012; Vallstedt and Kullander, 2013). Cross-midline inhibition relies on a dual inhibitory pathway (Figure 3A) composed mainly of V0-derived CIN and comprising: (i) a group of dorsal inhibitory CIN (V0_D_) that project monosynaptically to contralateral motor neurons; and (ii) a group of ventral glutamatergic CIN (V0_V_) which provide indirect inhibition via multisynaptic connections with ipsilateral inhibitory interneurons such as Renshaw cells (RC) and Ia inhibitory interneurons (Moran-Rivard et al., 2001; Pierani et al., 2001; Lanuza et al., 2004; Goulding, 2009; Kiehn et al., 2010). This system allows contralateral silencing during left-right alternation, in a frequency-dependent manner (Talpalar et al., 2013). In contrast, an excitatory pathway (Figure 3B) composed of glutamatergic CIN derived from V3 progenitors and projecting directly to contralateral motor neurons provides support for left-right synchrony (Zhang et al., 2008; Rabe et al., 2009; Borowska et al., 2013). This organization has been described in rodents (Quinlan and Kiehn, 2007; Restrepo et al., 2009) and cats (Jankowska et al., 2009). Additionally, ipsilaterally projecting interneurons are key components of multisynaptic pathways that provide indirect cross-midline inhibition and, as such, also participate in left-right alternation (Crone et al., 2008, 2009).

**Figure 4:**
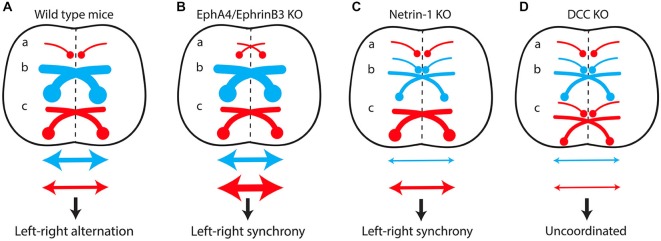
**The excitatory/inhibitory balance over the midline in the spinal cord determines the lateralization of motor output. (A)** In wild-type mice, predominant recruitment of the dual-inhibitory pathway at low locomotor frequencies produces cross-midline inhibition resulting in left-right alternating activity. A switch from alternating to synchronous left-right activity results from increased cross-midline excitation due to the formation of “pseudo-commissural” excitatory neurons (a) in EphA4/EphrinB3 knockout mice **(B)** or to misguidance of several populations of inhibitory commissural interneurons (b) in Netrin-1 knockout mice **(C)**. Conversely, the loss of both inhibitory (b) and excitatory commissural interneurons (c) in DCC mutants **(D)** produces uncoordinated left-right activity. (a) Excitatory ipsilateral interneurons. (b) Inhibitory commissural interneurons. (c) Excitatory commissural interneurons. Red arrow, cross-midline excitation; Blue arrow, cross-midline inhibition.

Mutant mice with commissural axon guidance defects have been critical for studying the spinal locomotor circuitry (Figure 4). Spinal CIN cross the midline at the floor plate, a structure located in the ventral spinal cord that secretes several molecules such as Ephrin-B3 and Netrin-1 involved in commissural axon guidance (Nawabi and Castellani, 2011). EphA4 and Ephrin-B3 knockout mice both have a hopping-gait phenotype (Dottori et al., 1998; Kullander et al., 2001b; Yokoyama et al., 2001). Fictive locomotion was studied with isolated spinal cords from EphA4 and ephrin-B3 null mutants aged between post-natal day 0 (P0) and P5, a period when the CST has not yet reached the lumbar spinal cord (Gianino et al., 1999). A switch from left-right alternating activity to synchronous activity was observed, together with an increased number of CIN in the ventral spinal cord. Reinforcement of cross-midline inhibition by GABA/glycine uptake blockers completely reversed this effect (Kullander et al., 2003). It was postulated that EphA4 is expressed in a population of excitatory interneurons projecting ipsilaterally, and that loss of EphA4 or ephrin-B3 leads to aberrant midline crossing of this population, resulting in “pseudo-commissural” excitatory connections. This would push the excitatory/inhibitory balance over the midline towards excitation (Figure 4B). In keeping with this hypothesis, specific deletion of EphA4 in the spinal cord or in glutamatergic interneurons is sufficient to induce a hopping gait both *in vivo* and *in vitro* (Borgius et al., 2014).

Netrin-1 knockout mice lack several inhibitory CIN populations, whereas their excitatory CIN are unaffected (Rabe et al., 2009). The inhibitory/excitatory balance over the midline is therefore shifted toward excitation, resulting in synchronous left-right locomotor activity *in vitro* (Figure 4C; Rabe et al., 2009). Surprisingly, suppression of the expression of DCC, the Netrin-1 receptor, leads to a different phenotype. DCC knockout mice exhibit uncoordinated left-right activity *in vitro*, reflecting the preservation of the excitatory/inhibitory balance over the midline, due to the loss of both inhibitory and excitatory CIN populations (Figure 4D; Rabe Bernhardt et al., 2012).

A hopping gait has also been described in Nkx mutant mice (Holz et al., 2010). Nkx transcription factors are involved in the development of the floor plate. The misguidance of V0 and dI6 CIN might be responsible for the phenotype of Nkx mutant mice (Holz et al., 2010).

Lateralization of motor output between the two sides of the spinal cord during stereotypic locomotion mainly relies on the excitatory/inhibitory balance over the midline. Recruitment of inhibitory pathways results in cross-midline inhibition and left-right alternation, whereas recruitment of the excitatory pathway results in a shift toward excitation and left-right synchrony. Supra-spinal control and descending pathways (CST in mammals, reticulospinal tracts in non mammalian vertebrates) do not participate in stereotypic left-right alternation but rather contribute to motor lateralization during voluntary movements and adaptive locomotion.

## Conclusion

The study of human “mirror movements” and rodent “hopping gait” reveals analogous mechanisms underlying the generation of asymmetric movements. Lateralized activation of the brain or spinal cord is first achieved through contralateral silencing by cross-midline inhibition. In the brain, this inhibition relies on excitatory neurons of the transcallosal tract that connect to inhibitory interneurons in the receiving hemisphere, while in the spinal cord both direct and indirect inhibition is involved during locomotion. Unilateral transmission of these activations results in lateralized motor output. When commissural axon guidance is compromised during development, the formation of projections to both sides of the spinal cord results in bilateral motor output. In mice, the formation of aberrant crossed excitatory connections in the spinal cord induces a hopping gait, while abnormal guidance of the CST in humans results in mirror movements.

## Author’s Role

QW, ID, CG and ER drafted the manuscript. QW, CG produced the figures. QW, ID, CG and ER critically reviewed the manuscript.

## Conflict of Interest Statement

The authors declare that the research was conducted in the absence of any commercial or financial relationships that could be construed as a potential conflict of interest.
